# Embedding Physical Activity in the Heart of the NHS: The Need for a Whole-System Approach

**DOI:** 10.1007/s40279-016-0488-y

**Published:** 2016-03-04

**Authors:** Helen Speake, Robert J. Copeland, Simon H. Till, Jeff D. Breckon, Steve Haake, Oliver Hart

**Affiliations:** Centre for Sport and Exercise Science, Sheffield Hallam University, Collegiate Hall, Collegiate Campus, Sheffield, S10 2BP UK; Sheffield Teaching Hospital NHS Trust, Royal Hallamshire Hospital, Sheffield, UK; Centre for Sports Engineering Research, Sheffield Hallam University, Sheffield, UK; Sloan Medical Centre, Sheffield, UK; National Centre for Sport and Exercise Medicine, Sheffield, UK; Centre for Health and Social Care Research, Sheffield, UK

## Abstract

Solutions to the global challenge of physical inactivity have tended to focus on interventions at an individual level, when evidence shows that wider factors, including the social and physical environment, play a major part in influencing health-related behaviour. A multidisciplinary perspective is needed to rewrite the research agenda on physical activity if population-level public health benefits are to be demonstrated. This article explores the questions that this raises regarding the particular role that the UK National Health Service (NHS) plays in the system. The National Centre for Sport and Exercise Medicine in Sheffield is put forward as a case study to discuss some of the ways in which health systems can work in collaboration with other partners to develop environments and systems that promote active lives for patients and staff.

## Key Points

**Table:** 

Solutions to the global challenge of physical inactivity have tended to be top-down, focusing on individual-level behaviour change.
To see population-level change in physical activity, a wider focus and multidisciplinary perspective is needed.
System-wide approaches present particular challenges for health systems and a new set of research questions.
Attempts to meet the challenge of physical activity by using systems thinking and user-centred design are explored from the perspective of one UK city (Sheffield).

## The Widespread Benefits of Physical Activity

The evidence supporting the personal and societal benefits of physical activity (PA) is now wide ranging and unequivocal. For individuals, PA is associated with improved mental and physical health and wellbeing [1] and prevention of non-communicable diseases (NCDs) [2, 3]. Societal benefits include a reduced burden on health and care services, active workplaces with reduced sickness absence and increased productivity, reduced effects of air pollution associated with increased active travel, and greater social capital and community spirit [4]. There are clear economic benefits; it is estimated that a 1 % reduction in inactivity could save £1.2 billion [5]; an 8-fold increase in cycling alone could save the UK National Health Service (NHS) £17 billion over 20 years [6]. Nevertheless, levels of PA are low and have remained relatively stable [7], with little impact demonstrated on improving population health and reducing inactivity.

## Current Efforts to Influence Physical Activity Behaviour

While concerns regarding the consequences of physical inactivity have grown over the past few decades [8], efforts to understand how health behaviour is influenced have also made significant advances. Taxonomies have been established highlighting the variety of strategies that can be employed to change behaviour, including PA [9]. Policies and interventions have tended to focus on individual psychological and cognitive–behavioural approaches, such as improving motivation [10, 11] to promote PA. While this evidence should not be disregarded, the downside of this approach has been that the resulting evidence base for PA behaviour change is dominated by interventions that affect the smallest number of people; far less is known about how to create population-level change, particularly in PA [12].

In reality, PA behaviour appears to be much more complex than simply being determined by personal agency. Social ecological models outline multiple levels of influence, including individual, interpersonal, environmental and policy factors [13]. Rather than being consigned to ‘contextual’ variables, these influences are considered to be multidirectional and dynamic [14]. These models suggest that motivating PA without removing barriers in the social or physical environment is likely to be ineffective [15, 16]. Indeed, advances in technology, increased desk-based employment, infrastructure designed around car use (and not walking and cycling), and the ubiquitous use of computers and television viewing in the home are all social/environmental factors that have had a negative effect on PA [17], largely through unconscious processing of behaviour. In addition, engineering PA out of daily life has not only reduced PA but also increased sedentary behaviour, which growing evidence now demonstrates is a risk factor for NCDs in its own right [18]. Social inequality in PA is evident in communities and environments, with low activity, poor health and wellbeing being associated with multiple indicators of deprivation [19]. For example, studies have found that poorer availability and accessibility of recreational facilities correlate with lower PA in both adults [20] and children [21].

As environmental influences on behaviour are increasingly recognised, phrases such as ‘nudging’ and ‘choice architecture’ have been coined to describe the strategies that policy makers, public organisations and private organisations are using to alter environments to influence behaviour. Public spaces and workplaces are being purposely redesigned to encourage and facilitate PA [22–24], and active living is promoted in European cities through urban design concepts such as ‘cyclability’ and ‘walkability’. Often these schemes are motivated by strategies to tackle rising obesity [25]—a key policy concern for many governments and municipalities, but one that overlooks physical fitness as the key determinant of good health. However, just as individual approaches alone may not be sufficient, environmental changes in isolation do not necessarily work in terms of promoting activity. For example, attempts to reduce sedentary behaviour in workplaces by providing sit–stand desks have been ineffective in maintaining changes in behaviour beyond 6 months [26], and a recent review of interventions in urban green spaces concluded that multifaceted programmes are more likely to impact on PA than changes to the built environment alone [27].

The wide-ranging social and economic benefits of increasing PA, coupled with models and interventions that identify multiple levels of influence, suggest that to move the agenda on PA forward, a multidisciplinary approach is needed.

## Physical Activity Promotion in the NHS

Health professionals have long been considered well placed to tackle what some have called the physical inactivity ‘pandemic’ [28, 29]. A recent review of National Institute for Health and Care Excellence (NICE) guidelines found that PA was recommended for almost 40 different health conditions [30]. In fact, any search of the published literature will produce an array of PA interventions across the spectrum of mental and physical health. The purpose of PA in these programmes varies, ranging from prevention to treatment and adjunct therapy, and then to rehabilitation, recovery and secondary prevention.

The diversity in the aims and outcomes of programmes and interventions, along with local commissioning of NHS services, has made it difficult to demonstrate their influence on PA at a population level. Even so, the data available, such as audits and reviews of cardiac rehabilitation programmes and exercise referral schemes, indicate that PA interventions are managed and delivered inconsistently across the UK [31, 32]. This leaves us with little proof that PA is being comprehensively promoted or that current efforts are having any sustainable impact on public health.

An evidence base has been built up biased towards controlled trials, which make a compelling case for the benefits of PA for a wide range of health issues, and, in this context, it is easy to see why a ‘prescription’ model of exercise medicine based on a biomedical approach has dominated. However, current systems for prescribing PA do not necessarily account for the complexity of the behaviour, nor its multilevel influences, since health promotion interventions have also followed the trend of targeting individual and interpersonal factors [33]. Poor adherence to taking regular medication has often been highlighted as a concern in the control of long-term conditions—for example, hypertension [34]. Arguably, adherence to a more complex PA prescription is even less likely. The NICE guidance [35] suggests that recommendations should be interpreted within a context of other interventions, including changes to the physical environment and other local strategies. A lack of knowledge on how to translate that suggestion into practice has perhaps prevented it from happening.

In PA promotion, the NHS has concentrated on relatively short-term, resource-intensive efforts, such as exercise referral schemes previously recommended by NICE [36]. However, evidence suggests that such approaches might not be cost effective [32] or feasible for the NHS to implement and deliver, with health professionals in primary care admitting that they adapt and modify elements of PA pathways because of lack of time and capacity, resorting to making subjective judgments to screen only those patients they perceive as suitable [37, 38]. Ultimately, the NHS may not have the money or the time to provide the requisite level of treatment fidelity in one-to-one behaviour change counselling that is considered a major factor in its success [39], and while these approaches work for particular individuals, a review of interventions worldwide indicates that they have modest impacts on maintenance of PA over the longer term [40]. With doubts existing over the effectiveness of current programmes, Simon Stevens, NHS England’s Chief Executive, placed a radical upgrade in prevention and public health as central to the sustainability of the NHS in his *Five Year Forward View* [41]. The NHS needs to try something different.

## A Design-Led, Person-Centred Approach

The term ‘intervention’ implies an element of interference or intrusion, which might not necessarily be invited or welcome. The traditional medical model places patients in the position of recipients, with health professionals assumed to know ‘what is best for them’, but in the case of PA, involving a complex interplay of human and environmental factors, this appears not to be enough.

Research into the public’s views on PA is surprisingly rare given the number of interventions trialled, but it offers insights into why ‘carrot and stick’ approaches, such as merely giving advice about the benefits of PA or the health costs of inactivity, have been ineffective in changing population PA behaviour [42]. Indications of what works for recipients of current interventions to promote PA in the NHS are being neglected. For example, assessment of the social environment created within exercise referral schemes is not traditionally included in key indicators and standard evaluation protocols, yet qualitative research indicates that this can significantly impact upon patient experience, attendance and adherence [43]. Studies into downloads of health apps in recent years have shown that people do not necessarily choose apps that are ‘evidence based’ [44, 45], and while these apps might not meet scientific standards in terms of demonstrating effectiveness, there are nevertheless important lessons to be learned about what influences their appeal among members of the public that could be translated into exercise medicine.

Recent reviews have criticised the ‘top-down’ approach to health service design, led by government initiatives, claiming that this undervalues the exploration of genuine needs and problems [46]. Evidence suggests that involving patients is critical for lifestyle change, so that interventions are aligned to patients’ real needs. At the same time, involving health professionals in the development of interventions means that they are more likely to adopt new protocols and that programmes will be fit for their purposes [47–49]. Rather than coaxing people to be more physically active, we should be actively involving them in the design of programmes that will have a mutual level of desirability and benefits. Integrated, person-centred care that values patients’ control of their health and is built on principles of holistic wellness and prevention is also at the heart of the NHS vision for the future [41].

Growing awareness of the ‘challenge’ to ensure that products and services designed to support change meet the needs, wants and expectations of those delivering and receiving them has led to increasing application of design-led approaches in health and public services [50, 51]. Mindsets and techniques used by private-sector design organisations—including journey mapping, rapid prototyping and iteration—are now being successfully applied at all levels of the public sector, from service improvement through to government policy [52]. Research using design thinking has helped to redefine healthcare problems and facilitate innovative solutions by starting from a position of empathy with the patients, carers and health professionals who ultimately determine the relative success or failure of services.

## Design-Led, Person-Centred and Multilevel Interventions in the NHS

There is already evidence that this approach can work in healthcare. Design-led research based on observation of patients, staff and everyday protocols in an accident and emergency (A&E) environment has generated positive changes in environments, processes and individual interactions, including better layout, better signage, clearer information and modified arrival and waiting processes [53]. Not only has this led to improved patient experiences and reduced aggression towards frontline staff [53], but also project evaluations report £3 worth of benefits for every £1 spent [54]. Involving patients directly in the redesign and refurbishment of low-secure mental health units, using a ‘serious gaming’ approach, has also provided valuable insight into the needs of otherwise difficult-to-engage groups and has led to practical service improvements [55].

Ecological approaches have achieved success when targeted at staff within the NHS too. A multicomponent workplace wellness intervention assessed over a 5-year period, which included health campaigns, provision of facilities and health promotion activities, was associated with positive effects on numbers of staff meeting PA guidelines, more active travel and a reduction in perceived barriers to PA [56]. This is promising given that connections have been drawn between positive staff working experiences and patient experiences [57], as well as the impact of health professionals’ personal health beliefs and behaviours on their tendency to promote PA [48]. Yet, despite these encouraging examples, environmental approaches towards promotion of PA among NHS patients have not been widely tested.

Multilevel interventions for active living require cooperation among professionals from many disciplines [42, 58]. Considering that the majority of environments that influence PA behaviour (e.g. home, work, school, neighbourhoods, transport [59]) are outside the control of health professionals [16], there is a good case for creating opportunities that facilitate increased collaboration and partnership working with other professionals.

## Towards a New Research Agenda

The challenge for professionals from all disciplines is to create the conditions for change across the entire system of PA. Recent reports have highlighted cities across the world as good practice examples of how PA can be made easier [60], and there is no doubt that it can be improved through interventions in multiple domains. More sharing of knowledge is needed by experts from different roles and a strategy for bringing together what is known about social, environmental and individual behaviour change to understand how to bring ecological models from theory into practice.

An initiative by UK innovation experts Nesta recently highlighted the importance of ‘realising the value’ in healthcare [61]. Translated into this context, there is a need to understand the particular value of PA for multiple stakeholders, to bring together a system-wide plan of action. Importantly, understanding the impact of such change on tackling health inequalities means that future research should aim to gather evidence that reflects real-world settings and is representative of the wider population. There are good practice examples of PA promotion across the NHS and other health systems internationally. More practice-based evaluation of how, when, why and for whom these work is needed.

From a health system point of view, several questions need to be explored:

- What is the NHS role within a system-wide approach to promoting PA? How do we integrate NHS interventions into a multidisciplinary agenda?
- How effective are ecological interventions that go beyond individual behaviour change in an NHS/healthcare context?
- How can the NHS develop systems capable of promoting PA among patients that also accommodate its complexity and multilevel influence?
- Do patients view PA as individually determined, or do they recognise a wider system of influences?

## National Centre for Sport and Exercise Medicine Sheffield: A Case Study

A recent International Olympic Committee (IOC) consensus statement recommended the establishment of specialist centres where existing evidence can be integrated with user-centred design, to develop sustainable and effective programmes that promote PA for the prevention of NCDs [62]. While there are several positive examples of programmes to tackle PA in healthcare—the centres of the Exercise is Medicine^®^ global health initiative being of notable consideration [63]—the following section considers one case study of a UK city adopting systems thinking and user-centred design to promote PA.

The National Centre for Sport and Exercise Medicine (NCSEM), currently being established in Sheffield, is one of three UK sites funded as part of the London 2012 Olympic legacy. Sheffield aims to reduce the burden of NCDs by improving the physical fitness of Sheffield’s residents through the propagation of a city-wide philosophy whereby being physically active at home, while commuting, at work, in NHS care and in recreation time is the norm rather than the exception.

The following section explores several key principles underpinning the NCSEM work, which attempts to extend current practice and explore new ways of working. The NCSEM model will allow these principles to be tested and evaluated for their contribution to the strategy to improve the PA culture of Sheffield.

### Commissioners and Providers of Health Services as Key Partners in a ‘Whole-City’ Approach

NCSEM is a part of a city-wide strategy, under the banner ‘Move More’. Sheffield will target the whole population, creating environments and supporting individuals and communities to engage in enough PA to be healthy (and sustain it). This will include everything from improving grass-roots sport in schools to supporting people back into work through increasing their PA. To give this whole-system approach the best chance of success, all of the partners who can make it happen have invested in the strategy—including town planners, health care professionals, the fitness industry, business executives, teachers, architects, academics and community leaders. Of perhaps most importance will be the involvement of patients and local people whom the strategy is trying to support.

### Raising Standards of Evidence in Existing Interventions Will Help to Identify Active Ingredients

Institutions such as NICE have previously noted the difficulty in evaluating health promotions because of a lack of good-quality evidence [36]. NCSEM Sheffield is attempting to gather evidence on what works to improve the PA of Sheffield’s residents by raising the quality of evidence being collected from interventions already taking place across the city. Searching for the most robust levels of evidence means that often the evidence available comes from studies with small, convenience or volunteer samples. It has been suggested that by relying on such evidence, we are at risk of designing interventions that worsen health inequalities rather than reducing them [12]. NCSEM Sheffield is attempting to raise standards of evidence [64] while also integrating the ‘soft evidence’ that might be overlooked by a purely reductionist approach, but that ultimately might better capture the human elements of PA behaviour, which we need to understand for new programmes to work.

### Putting Members of the Public at the Forefront of Designing Programmes to Promote Physical Activity Will Make the Programmes More Desirable and Feasible

NCSEM has a strong, multidisciplinary leadership but aims to embed PA from the grass roots up. Crucially, patients are not going to be ‘subjects’ of inspiration for design [65] but will be active partners, with research underway to co-design PA pathways involving patients and frontline health professionals. Rather than asking “How can we make patients more active?”, this means asking “How can PA help our patients, and how can we support them to make this happen?” It will involve identifying patients’ health-related needs and aspirations, and designing a PA pathway that facilitates and supports these, while also recognising the training and resources needed by health professionals to appropriately deliver that support. The design methodology encourages an iterative approach during development of the pathway, evaluating both appeal and practicality along the way and making adjustments as necessary.

### Re-incentivising Health Services to Identify and Meet Patient-Centred Outcomes Will Help Promote Physical Activity

The recent *Designed to Move: Active Cities* report shows that asking people what they need to be able to move more has been most successful [60]. In support of this whole-system approach, Sheffield Clinical Commissioning Group has recommissioned musculoskeletal (MSK) services (those specialities that provide MSK care, including chronic pain management, community physiotherapy, metabolic bone disease management, orthopaedics, podiatry, rheumatology, and sport and exercise medicine) to focus more on patient outcomes than volumes of service delivery. The old payment-by-results system rewarded hospitals for delivering activity; the new contract will reward achievement of patient-determined outcomes. Local co-production of outcomes has highlighted a wish for better prevention, promoting PA and a person-centred approach. Consequently, the new MSK system will be designed and incentivised to include increasing PA levels as a core outcome.

### Changes to Physical NHS Environments Will Help to Promote Physical Activity

The IOC statement calls for health services to work in communication with the fitness and wellness industry [62], which, although not built on principles of health or disease prevention, possesses relevant facilities, distribution networks and expertise. With this in mind, one of the unique programmes of NCSEM Sheffield is a ‘Hub and Spoke’ model, which co-locates NHS clinical teams, researchers and patients in community-based leisure facilities, making it easier to promote and undertake ‘PA as medicine’.

A wealth of knowledge on behaviour change at the individual level now exists, and controlled research has demonstrated that the quality and fidelity of delivery affect its effectiveness. What is not clearly understood is the proportion of quality that is defined by the environment in which that delivery takes place. The creation of high-profile facilities that combine health delivery with leisure and sport facilities will aim to help change the culture of the city through promotion of the ethos that maintenance of good health through PA is a normal part of life for people living in Sheffield. Furthermore, the NCSEM facilities will create an opportunity to shape the environment for people with health needs that can be improved/treated through increasing PA. The creation of a number of sites that can be linked together (hence Hub and Spoke) will also facilitate a network of good practice, and it is the intention that the NCSEM facilities will support NHS aspirations to bring high-quality services closer to home [66]. The purpose of the NCSEM Hub and Spoke model is therefore simple: to ‘make it easier’ for people to choose PA as part of an NHS care pathway as well as in their daily lives.

## Conclusions

Green and Glasgow previously suggested that “if we want more evidence-based practice, we need more practice-based evidence” [67]. NCSEM Sheffield and the accompanying city-wide ‘Move More’ strategy present an opportunity to observe which aspects of the environment influence PA and how best to implement particular strategies. The NCSEM Sheffield ethos is consistent with aspirations for the future shape of the NHS described in the Five Year Forward View: supporting people to remain healthy through preventative measures, getting the best possible value from NHS spending and investing in ways of providing more integrated and collaborative care [68]. The NHS needs to adapt and evolve to a changing population profile, including an aging population and emerging and increasing burdens of disease, if it is to stand any chance of meeting current and future ill-health demands and remaining viable. As noted previously, such challenges require innovation and novel ways of thinking and working, extending relationships and networks beyond traditional boundaries, as well as better translation of the valuable, robust evidence that already exists into practice. The NCSEM model is an ambitious example of the NHS embracing innovation and working in partnership to take on a complex issue.
